# Author Correction: Large-scale mass wasting in the western Indian Ocean constrains onset of East African rifting

**DOI:** 10.1038/s41467-020-18395-8

**Published:** 2020-08-28

**Authors:** Vittorio Maselli, David Iacopini, Cynthia J. Ebinger, Sugandha Tewari, Henk de Haas, Bridget S. Wade, Paul N. Pearson, Malcolm Francis, Arjan van Vliet, Bill Richards, Dick Kroon

**Affiliations:** 1 Department of Earth and Environmental Sciences, Life Sciences Centre, Dalhousie University, Halifax, NS B3H 4R2 Canada; 2 Dipartimento di Scienze della Terra, dell’Ambiente e delle Risorse, Università degli Studi di Napoli Federico II, Naples, 80126 Italy; 3 School of Geosciences, University of Aberdeen, Aberdeen, AB24 3FX United Kingdom; 4 Department of Earth and Environmental Sciences, Tulane University, New Orleans, LA 70118 United States of America; 5 WesternGeco, Gatwick, RH6 0NZ United Kingdom; 6 National Marine Facilities, Royal Netherlands Institute for Sea Research and Utrecht University, Utrecht, The Netherlands; 7 Department of Earth Sciences, University College London, London, WC1E 6BT United Kingdom; 8 School of Earth and Ocean Sciences, Cardiff University, Cardiff, CF10 3AT United Kingdom; 9 Royal Dutch Shell, 2596 HR The Hague, The Netherlands; 10 School of GeoSciences, University of Edinburgh, Edinburgh, EH9 3FE United Kingdom

**Keywords:** Geophysics, Sedimentology, Tectonics

Correction to: *Nature Communications* 10.1038/s41467-020-17267-5, published online 10 July 2020.

The original version of this Article contained an error in the spelling of the co-author Malcolm Francis.

This has been corrected in both the PDF and HTML versions of the Article.

