# Supplementary material for: Large-scale mass wasting in the western Indian Ocean constrains onset of East African rifting
Source: Nat Commun. 2020 Jul 10;11:3456. doi: 10.1038/s41467-020-17267-5 (PMC7351987; doi:10.1038/s41467-020-17267-5)
Supplement: Supplementary file 1 — Supplementary Information [file 41467_2020_17267_MOESM1_ESM.pdf]

SUPPLEMENTARY INFORMATION

Large-scale mass wasting in the western Indian Ocean constrains onset of East African rifting

by Maselli et al.

Supplementary Note 1

Seismic facies characterization. The seismic profiles presented in Figures 3 to 6 of the manuscript highlight the post-Eocene stratigraphic succession of the Tanzanian margin. The post-Eocene shows different seismic facies and reflection configurations that are overall generated by sediment deposition in deep-water settings. The seismic facies are as follows (Supplementary Figure 1): Facies 1 is characterized by wavy reflections, both low- and high-amplitude that may reflect the presence of contourite drifts and bottom-current deposits[1,2]. Seismic facies 2 shows sedimentary packages with high-amplitude reflections (F2a), often intercalated with more chaotic units (F2b) and can be related to gravity-flow deposits (turbidites and debrites) along the axis of a slope channel[3,4]. Facies 3 shows continuous, low-amplitude and often wavy reflections, with reflection packages laterally thinning, and can be associated with channel-levee deposits[4,5]. Facies 4 consists of thick and high-amplitude reflections forming tabular to lens-shaped packages that can be associated with submarine fan deposits[6]. Facies 5 has high-frequency, low- to very low-amplitude, and parallel reflections that may represent hemipelagic sediments. The examples provided in Supplementary Figure 1 are extracted from the dataset available.

Supplementary Note 2

Depositional Units 1-13. In this section, we present a detailed description of each of the 13 Depositional Units (DUs) identified in the upper ~1000-m-thick stratigraphic interval (Supplementary Figures 2 and 3), which corresponds to ~45 million years. The units were identified by integrating seismic and Gamma-Ray (GR) data, the latter directly relate radioactivity changes to grain size variations[7].

The depth of each unit is reported as millisecond Two-Way Travel time (ms TWT) at the well. The units are bounded by seismic horizons associated with truncations and onlaps, interpreted as sequence boundaries, and horizons associated with onlaps or downlaps, interpreted as flooding surfaces. The sequence boundaries tie to sharp-based lithological contacts on the GR log, while the flooding surface

to ‘hot’ GR intervals or upward increases in GR (Supplementary Figure 3 and Figure 2 in the manuscript). Supplementary Figure 3 shows a close-up view of Line 2 (Supplementary Figure 2) across the drill site, with Well-1 trace and GR log superimposed in TWT using the check-shot survey. The GR log is displayed to the left of the well trace, while the interval velocities calculated from check-shot values are shown to the right of the well trace, with low velocity to the left (minimum values of 1,670 m s^{-1}) and high velocity to the right (maximum 3,900 m s^{-1}, near the bottom of the well). The 13 DUs also correspond to broad patterns in the interval velocity, although without a synthetic seismogram at seismic bandwidth it is not straightforward to analyse reflections at the half-cycle level in terms of specific interfaces or groups of interfaces. Unit 10, the Mafia mega-slide, was mapped throughout the study, while the other depositional units were interpreted near the well-tie and have not been extended regionally.

Beginning at the base of seismic Line 1 (Supplementary Figures 2 and 3), DU-1 (2,900-2,785 ms TWT at the well) is a ‘transparent’ seismic interval at its base (low amplitudes, low reflectivity, moderate continuity) but reflection amplitudes increase moderately upwards. It ties to a high GR, fine-grained, interval at the well (mudstone with minor silts) and increases in velocity upwards. This internal is interpreted as deep-water pelagic mudstones.

The overlying DU-2 (2,785-2,760 ms TWT) is conformable but relatively thin at the well and, seismically, it comprises three high-amplitude half cycles. On the GR, DU-2 is shalier than DU-1, with several velocity changes giving rise to higher reflectivity. Based on seismically observed thickness changes and reflection truncations it is interpreted to be partly eroded below DU-3.

DU-3 (2,760-2,730 ms TWT) contains regional horizon M1 and is interpreted to be erosional at both its base and top, as indicated by truncations and onlaps. The GR log indicates a sharp-based blocky interval, probably fine-grained sand or silty-sand that has moderate to high interval velocities.

DU-4 (2,730-2,715 ms TWT) is thin, but it has a classic sharp-based blocky to fining up sand-to-silt signature on the GR log. It has high velocity with a thin low velocity interval near its base. Up to 60 ms of erosion is visible east of the well (Supplementary Figure 3).

DU-5 (2,715-2,625 ms TWT) is a ~100-m-thick unit at the well, with a low amplitude / transparent seismic response and a GR log that fines upward from thin sands and silts near its base to mudstone, and then has a thin coarsening upward interval near its top. Despite being mudstone-on-mudstone at the well, the surface at the top of DU-5, which corresponds to horizon M2, has considerable erosional relief west of the well and is interpreted as a sequence boundary.

DU-6 (2,625-2,610 ms TWT) is a thin and high GR interval that increases in velocity upward. It thickens westward on seismic, displaying distinctive eastward downlap of high-amplitude reflections on to its basal horizon (Supplementary Figure 3).

DU-7 (2,610-2,585 ms TWT) continues a weak upward trend of increasing velocity and decreasing GR but the lower section of GR log is missing. Seismically it has a clear erosional top, and possibly an erosional base with weak-moderate amplitude and semi-continuous reflections between.

DU-8 (2,585-2,550 ms TWT) is a sharp-based unit, fining (and slowing) upward from its (sand) base on the GR log to a sharp silt-to-mud deepening at its top. Seismically, it has an erosional base and a downlap surface at its top.

DU-9 (2,550-2,495 ms TWT) comprises three thin mudstone-siltstone coarsening upward intervals on the GR log with an overall slowing upward signature (Figure 2 in the manuscript). Seismically, it comprises weak-moderate amplitude reflections downlapping onto a basal horizon.

DU-2 to DU-9 are interpreted as stacked deep-water lobes, locally reworked by distributary channels, that represent a phase of enhanced sediment supply compared to DU-1, and progradation of the margin.

DU-10 (2,495-2,392 ms TWT) is the Mafia mega-slide, bounded by a clear erosional surface at the base and a mounded and irregular reflection at its top.

DU-11 (2,392-2,275 ms) comprises two fine-grained coarsening upward intervals capped by a thin sharp-based fining upward unit. Seismically DU-11 is very distinctive: low- to high-amplitude sub-horizontal and conformable reflections onlap and infill the considerable topography at the top of the mega-slide.

DU-12 (2,275-1,949 ms TWT) is lumped as one depositional unit, with horizon M4 at its top. On the GR log, DU-12 comprises ~300 m of fine-grained sediments with at least ten coarsening upward sequences (see Figure 2 in the manuscript). Horizon M3 is encountered at a depth of 2,152 ms TWT.

Between the M4 and the sea floor, DU-13 (1,949-1,839 ms TWT) has limited GR (coarsening-up) and velocity data. It appears transparent seismically near its base, transitioning upwards into higher amplitude conformable to disconformable reflections with a strong water bottom reflection at its top.

DU-9 to DU-13 are interpreted as deep-water gravity driven deposits, such as turbidites, deposited in a more proximal position compared to the units below.

Supplementary Note 3

Sedimentation rates along the margin of the western Indian Ocean. Supplementary Figure 4 shows sedimentation rates in meters per million of years (m Myr^{-1}) calculated using the modern thickness of

the different stratigraphic intervals in Well-1, Well-2, and DSDP-241 offshore Somalia[8], and the chronology derived from nannoplankton. This approach underestimates sedimentation rates because it is based on modern compacted thickness and does not include loss of sediment at minor erosional surfaces.

For Well-1, sedimentation rate between 28 Ma (age of horizon M2) and 15 Ma (age of horizon M3), namely S_{rate}, is a mean value calculated as:

$$S_{rate}=(H_{M2\text{-}M3}-H_{MS})/T$$

where T= 13 Myr, $H_{M2\text{-}M3}$ is the thickness of the M2-M3 interval, equal to 474 m, and H_{MS} is the thickness of the Mafia mega-slide, equal to 100 m. The thickness of the Mafia mega-slide is removed from the total thickness as it can be considered an instantaneous event. Sedimentation rate is thus estimated at 28.8 m Myr^{-1}.

The mean sedimentation rate in the interval 28-15 Ma in Well-2, calculated using Supplementary Table 4, is 160 m Myr^{-1} if we include the two peaks of 424 m Myr^{-1} and 250 m Myr^{-1} (see Supplementary Figure 4), or 42 m Myr^{-1} without them. The two peaks represent a landslide deposit and a fast aggrading channel, so they can be removed from the calculation for evaluating the background sedimentation.

If we consider that at burial depth of ~2,000 m the compaction of sediment is about 30%[9], the mean sediment accumulation rates for de-compacted deposits in Well-1 and Well-2 for the interval 28-15 Ma are 37.4 m Myr^{-1} and 48 m Myr^{-1}, respectively. For the aim of this study, it is possible then to consider that the sediment accumulation rate between 28 Ma and 15 Ma in the slope area of the Tanzania margin is between ~29 m Myr^{-1} and 48 m Myr^{-1} (Supplementary Figure 4).

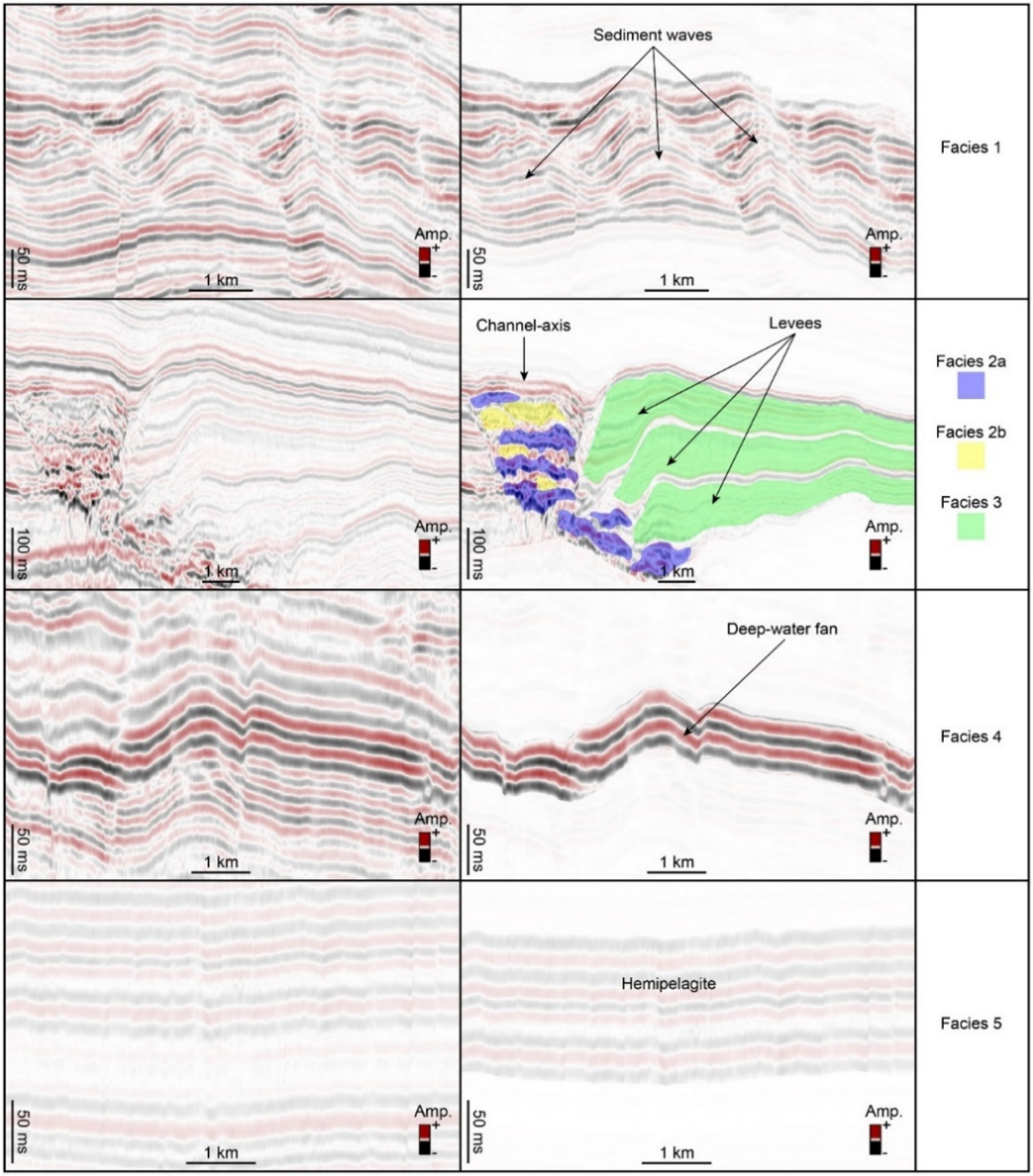

Supplementary Figure 1. Seismic facies characterization. Seismic facies characterization of the main slope depositional elements in the study area.

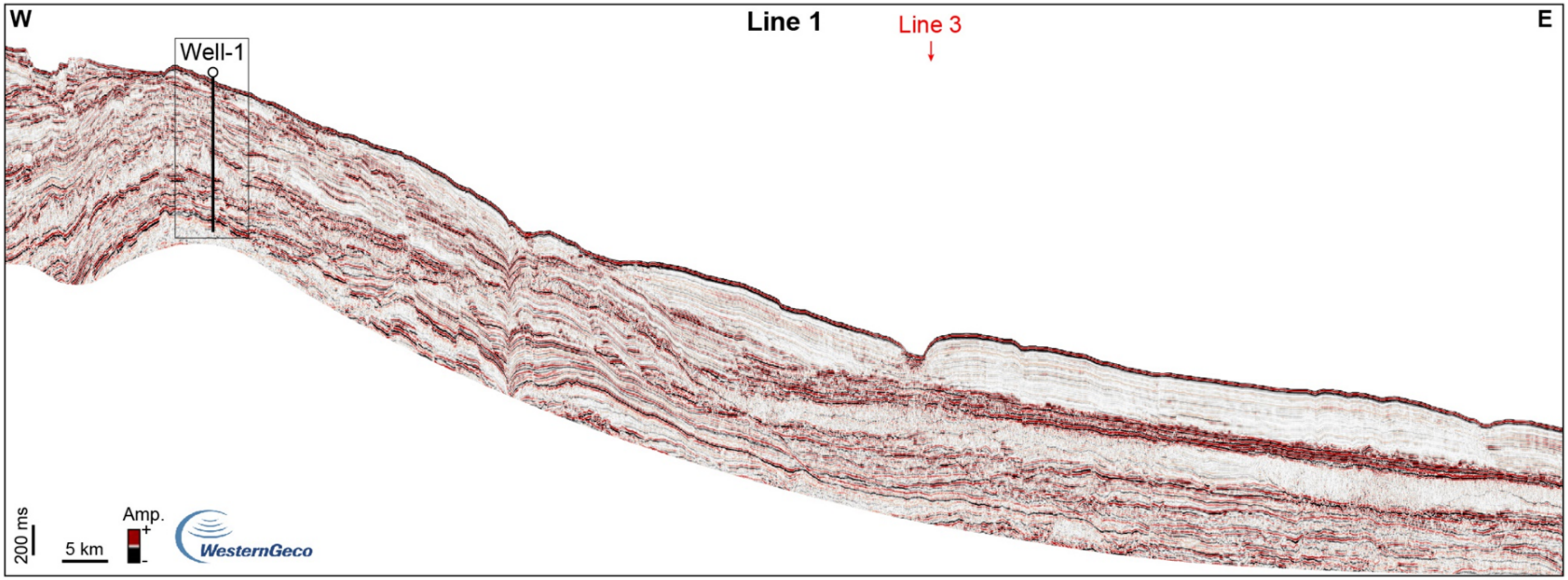

Supplementary Figure 2. Stratigraphy of the Tanzania margin imaged by a downslope-oriented seismic line crossing Well-1. Non-interpreted version of seismic Line 1 with location of Well-1. The black square is the detail presented in Supplementary Figure 3.

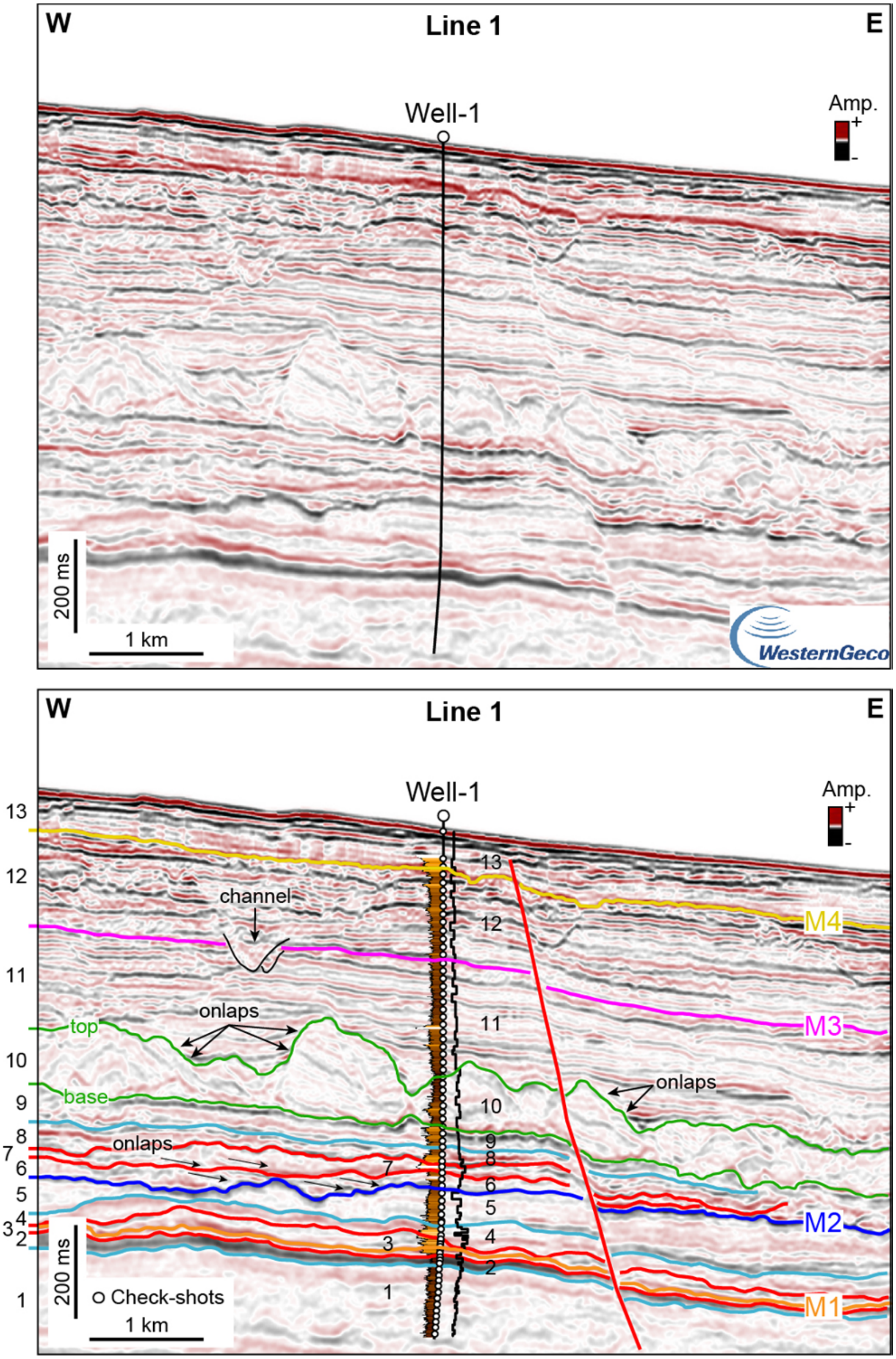

Supplementary Figure 3. **Detail of seismic Line 1 at Well-1.** Top: Non-interpreted version of seismic Line 1 with location of Well-1; Bottom: Interpreted seismic line with well trace, Gamma-Ray, check-shots marked by white circles, interval velocity, Depositional Units (1-13) discussed in Supplementary Note 1, and dated stratigraphic horizons (M1 to M4).

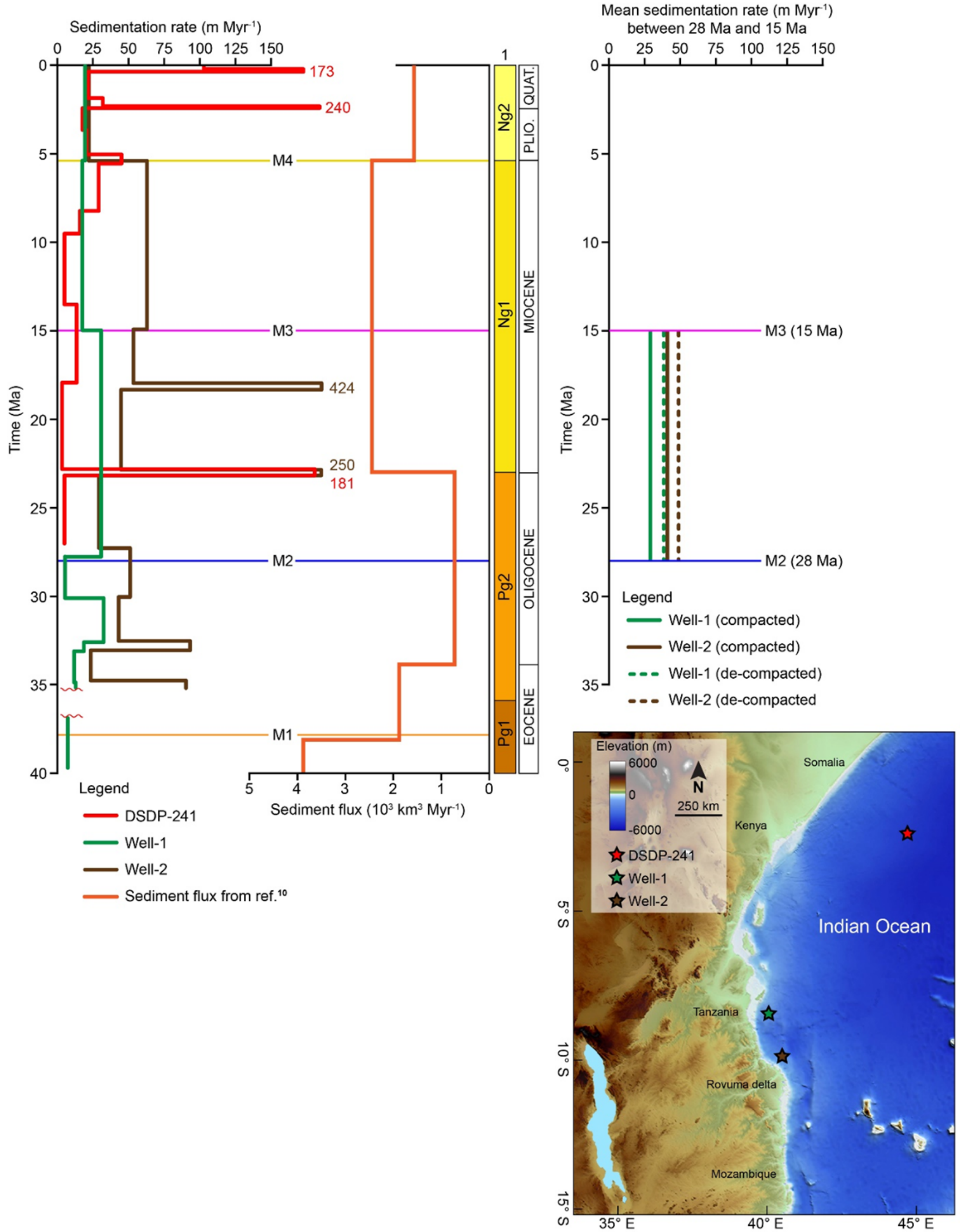

Supplementary Figure 4. **Sedimentation rates in the western Indian Ocean.** Sedimentation rates in the western Indian Ocean derived from three wells (Well-1, Well-2, and DSDP-241[8]) and sediment flux for the Tanzania margin (modified from ref.[10]). 1: Stratigraphic sequences from ref.[11].

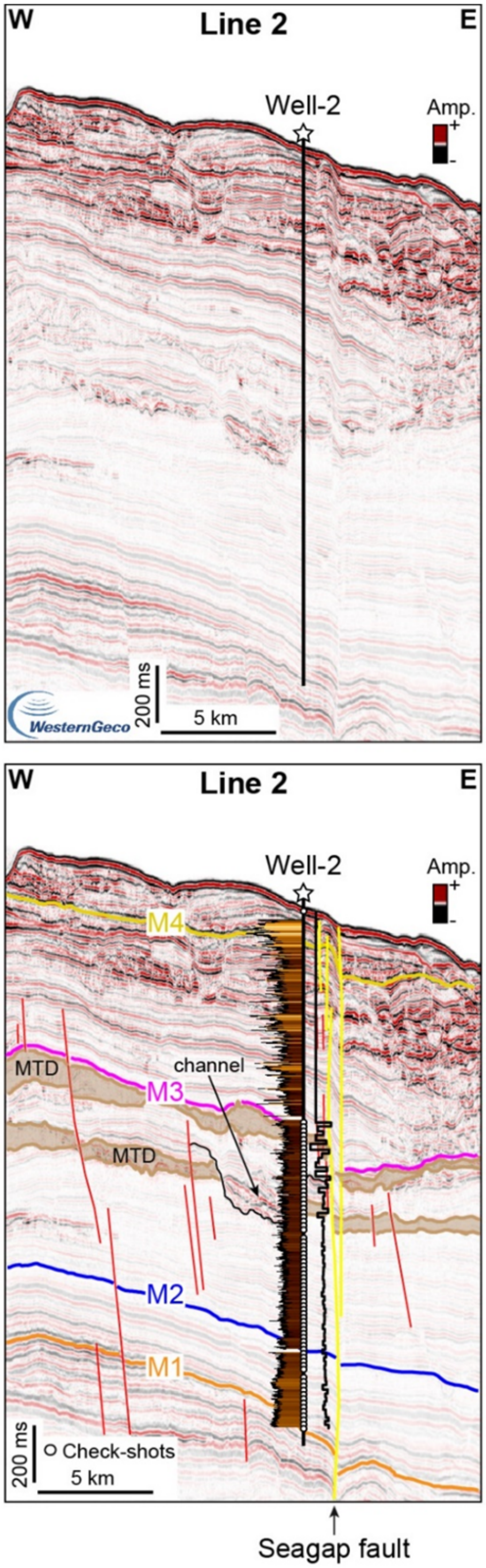

Supplementary Figure 5. **Stratigraphy of the Tanzania margin imaged by a downslope-oriented seismic line crossing Well-2.** Top: Non-interpreted version of seismic Line 2 with location of Well-2; Bottom: Interpreted seismic line with well trace, Gamma-Ray, check-shots marked by white circles, interval velocity, and dated stratigraphic horizons (M1 to M4).

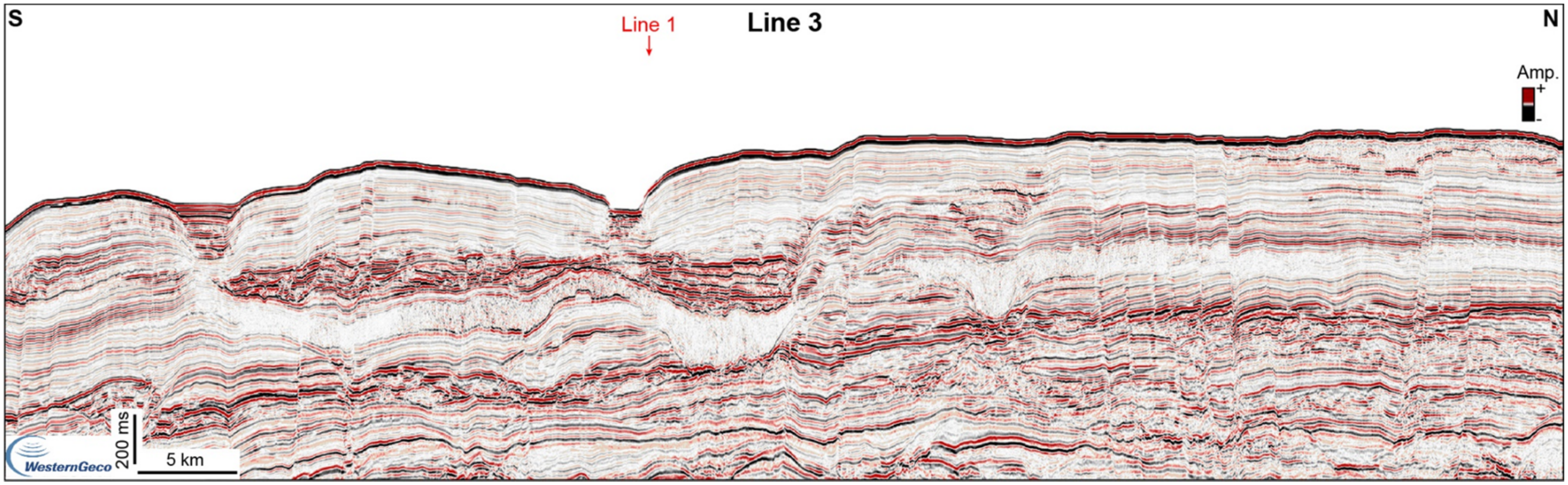

Supplementary Figure 6. Seismic line showing the along-strike variability of the Mafia mega-slide and bounding deposits. Non-interpreted version of seismic Line 3.

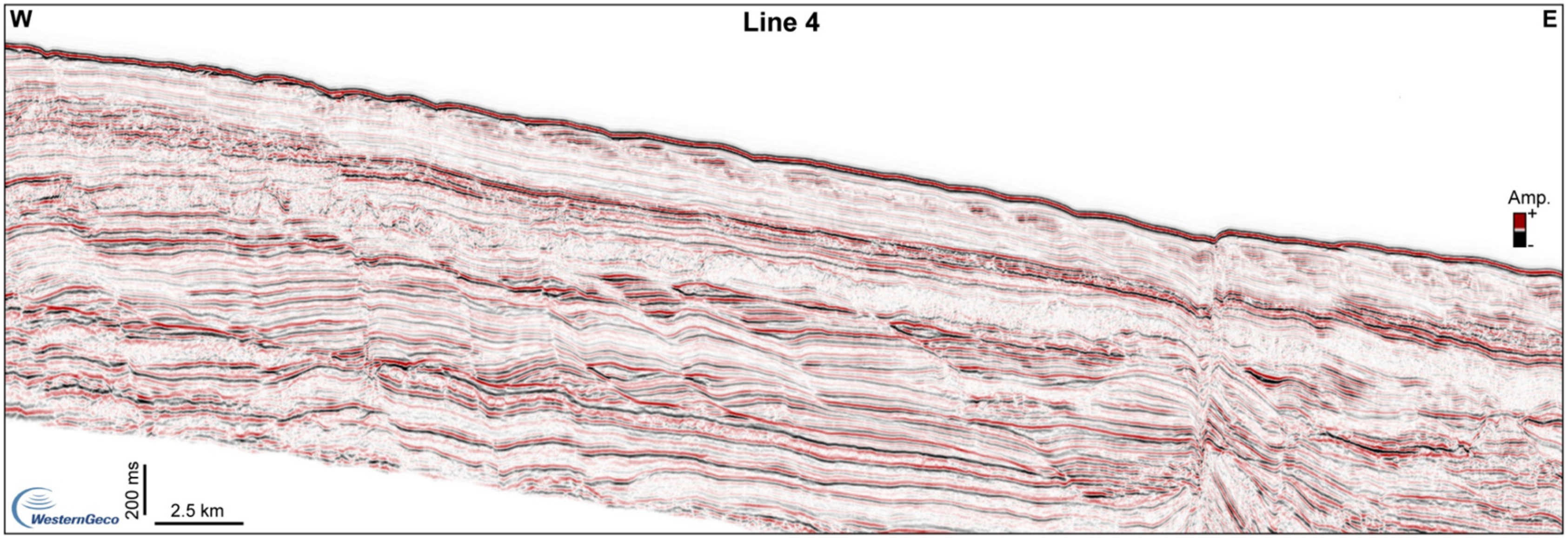

Supplementary Figure 7. Downslope-oriented seismic line across the Mafia mega-slide. Non-interpreted version of seismic Line 4.

Supplementary Table 1. **Micropaleontology of exploration Well-1.**

Depth (m)	Zone/Subzone	Event
2,270	?E16	TOP *Turborotalia cerroazulensis*
2,280	?E14-?E13	*Turborotalia cunialensis* (C)
2,290	E12 (reworked)	PRES *Orbulinoides beckmanni* (R)
2,300-2,350	E9-E8	TOP in situ *Igorina broedermanni*, *Morozovella aragonensis*, PRES in situ *Igorina anapetes*

<u>Abbreviations</u>
TOP: first downhole occurrence/extinction
PRES: single occurrence of a taxon
R: reworking
C: caving

The planktonic foraminiferal zones are from ref.[12]. Biostratigraphic data are provided by Royal Dutch Shell and Shell Tanzania. Depth is expressed in meters ssTVD.

Supplementary Table 2. **Nannopaleontology of exploration Well-1.**

Depth (m)	Zone/Subzone	Event
2,140-2,150	NP24	TOP (TNS) *Sphenolithus predistentus*, *Sphenolithus distentus*, *Sphenolithus ciperoensis*, *Calcidiscus protoannulus*, HRA *Triquetrorhabdulus carinatus*
2,160-2,230	NP23	PRES *Helicosphaera compacta*, TOP *Helicosphaera bramlettei*, BASE OCC/CMN *Sphenolithus ciperoensis*
2,240	NP22	INC *Sphenolithus predistentus* (ABN), BASE/HRA (CMN) *Sphenolithus distentus*, BASE *Cyclicargolithus abisectus*
2,250-2,260	NP21	PRES *Pemma spp*., HRA *Clausicoccus fenestratus*, TOP consistent *Helicosphaera compacta*, DEC *Sphenolithus distentus* (C), TOP further INC/SABN *Sphenolithus predistentus*
2,270	NP20-NP19	INC *Reticulofenestra stavensis*, BASE ABN *Sphenolithus predistentus*, TOP *Lanternithus minutus*
2,280	NP17 (upper)	PRES *Sphenolithus obtusus*, BASE *Reticulofenestra stavensis* (HRA/ABN), TOP INC *Lanternithus minutus*, *Reticulofenestra hesslandii* (SABN), TOP DEC *Sphenolithus predistentus* (CMN)
2,290-2,300	NP15c	INC *Zygrhablithus bijugatus*, BASE SABN *Reticulofenestra hesslandii*, TOP OCC/CMN *Sphenolithus radians*, TOP ACME *Discoaster barbadiensis*, *R. wadeae*, *S. spiniger*, *S. furcatolithoides*, *Pseudotriquetrorhabdulus inversus* (ABN), TOP *Calcidiscus pacificanus*, *Campylosphaera dela*, *S. perpendicularis*
2,310-2,350	NP15b	TOP *Chiasmolithus gigas*, *Blackites kilwaensis*, *Nannotetrina cristata*, DEC *Cyclicargolithus floridanus* (C)

<u>Abbreviations</u>
TOP: first downhole occurrence/extinction
BASE: last downhole occurrence/inception
OCC: occasional
CMN: common
ABN: abundant
SABN: superabundant
INC: downhole increase in abundance
DEC: downhole decrease in abundance
PRES: single occurrence of a taxon
C: caving
TNS: top not seen
HRA: high relative abundance

Nannofossil zonations are the NP zones of ref.[13]. Biostratigraphic data are provided by Royal Dutch Shell and Shell Tanzania. Depth is expressed in meters ssTVD.

Supplementary Table 3. **Micropaleontology of exploration Well-2.**

Depth (m)	Zone/Subzone	Event
2,380-2,440	M5	ABS *Orbulina* spp., PRES *Globorotalia archaeomenardii*
2,460-2,620	M5-M4	ABS *Globorotalia archaeomenardii*
2,640-2,720	M3-M2	TOP *Catapsydrax dissimilis*
2,740-2,820	Intra M2	TOP *Globigerinoides primordius*, *Dentoglobigerina selli*
2,840-3,020	M1a	TOP *Paragloborotalia nana*
3,040-3,140	O7-O6	BASE *Paragloborotalia pseudokugleri*
3,160-3,300	O6-O4	BASE *Globigerina angulisuturalis*, TOP *Dentoglobigerina galavisi*, *Dentoglobigerina pseudovenezuelana*
3,320-3,380	O3-O2	TOP *Dentoglobigerina galavisi*, *Dentoglobigerina pseudovenezuelana*, *Turborotalia ampliapertura*, *Turborotalia increbescens*, *Dentoglobigerina tripartita*
3,420-3,480	O1	TOP *Subbotina eocaena*
3,500-3,530	E16-E15	TOP *Turborotalia cerroazulensis*

Abbreviations
TOP: first downhole occurrence/extinction
BASE: last downhole occurrence/inception
PRES: single occurrence of a taxon
ABS: absence

The planktonic foraminiferal zones are from ref.[12,14]. Biostratigraphic data are provided by Royal Dutch Shell and Shell Tanzania. Depth is expressed in meters ssTVD.

Supplementary Table 4. Nannopaleontology of exploration Well-2.

Depth (m)	Zone/Subzone	Event
2,370-2,430	NN5	TOP *Sphenolithus heteromorphus*, *Helicosphaera perch-nielseniae*, TOP *Calcidiscus premacintyrei*
2,450-2,470	NN5-NN4	PRES *Orthorhabdus serratus*, TOP *Helicosphaera obliqua*, TOP *Discoaster petaliformis* (CMN) BASE CMN *Discoaster petaliformis*, TOP *Sphenolithus milanetti*, BASE *Coronocyclus nitescens* (elliptical)
2,490-2,610	NN4	BASE *Calcidiscus premacintyrei*, PRES OCC/CMN *Sphenolithus milanetti*, TOP *Reticulofenestra lockeri*, *Sphenolithus belemnos*
2,630-2,750	NN3	TOP *Hughesius tasmaniae*, BASE CMN *Sphenolithus heteromorphus*, TOP HRA *Sphenolithus belemnos*, BASE *Sphenolithus belemnos*
2,770-2,950	NN2	TOP *Triquetrorhabdulus carinatus*, TOP *Discoaster druggii*, TOP *Sphenolithus disbelemnos*, PRES *Helicosphaera mediterranea*, TOP HRA *Triquetrorhabdulus carinatus*, TOP INC recovery with TOP *Reticulofenestra lockeri*, *Coccolithus eopelagicus*, BASE consistent *Sphenolithus disbelemnos*, BASE *Discoaster druggii*
2,970-3,030	NN1	TOP *Sphenolithus delphix*, TOP *Sphenolithus calyculus*, PRES *Cyclicargolithus abisectus*, PRES *Sphenolithus ciperoensis* (R), TOP *Helicosphaera recta* (in situ), BASE *Sphenolithus delphix*
3,050-3,150	NP25	TOP *Sphenolithus ciperoensis*, TOP *Reticulofenestra stavensis*
3,170-3,290	NP24	TOP *Cyclicargolithus abisectus*, *Sphenolithus distentus*, INC *Sphenolithus ciperoensis*, *Helicosphaera recta*, TOP *Sphenolithus predistentus*, *Sphenolithus distentus*, BASE HRA *Triquetrorhabdulus carinatus*, TOP INC recovery with ABN *Sphenolithus predistentus*, CMN *Reticulofenestra stavensis*
3,310-3,400	NP23	TOP *Cyclicargolithus floridanus*, *Helicosphaera bramlettei*, *Discoaster tanii*, *Helicosphaera compacta*, BASE *Sphenolithus ciperoensis*, TOP *Discoaster ornatus*, *Sphenolithus pseudoradians*, *Helicosphaera seminulum*, *Reticulofenestra hillae*, *Sphenolithus radians*, *Discoaster barbadiensis* (R), *Discoaster saipanensis* (R)
3,410-3,450	NP22	TOP *Sphenolithus tribulosus*, BASE ABN *Reticulofenestra hillae*
3,460-3,490	NP21	TOP SABN *Lanternithus minutus*, CMN *Clausicoccus fenestratus*, PRES ABN *Helicosphaera robinsoniae*, TOP *Coccolithus formosus*, *Isthmolithus recurves*, BASE SABN *Lanternithus minutus*
3,500-3,530	NP20-NP19	BASE *Helicosphaera euphratis*, BASE CMN *Clausicoccus fenestratus*, TOP consistent *Discoaster saipanensis*, TOP *Discoaster barbadiensis*, TOP CMN *Calcidiscus? protoannulus*, BASE *Isthmolithus recurvus*, TOP *Cribrocentrum reticulatum*

Abbreviations

TOP: first downhole occurrence/extinction
BASE: last downhole occurrence/inception
OCC: occasional
CMN: common
ABN: abundant
SABN: superabundant
INC: downhole increase in abundance
DEC: downhole decrease in abundance
PRES: single occurrence of a taxon
R: reworking
HRA: high relative abundance

Nannofossil zonations are the NP zones of ref.[13]. Biostratigraphic data are provided by Royal Dutch Shell and Shell Tanzania. Depth is expressed in meters ssTVD.

Supplementary References

1. Rebesco, M., Hernández-Molina, F.J., Van Rooij, D. & Wåhlin, A. Contourites and associated sediments controlled by deep-water circulation processes: state-of-the-art and future considerations. Marine Geology 352, 111–154 (2014).
2. Fonnesu, M., Palermo, D., Galbiati, M., Marchesini, M., Bonamini, E. & Bendias, D. A new world-class deep-water play-type, deposited by the syndepositional interaction of turbidity flows and bottom currents: The giant Eocene Coral Field in northern Mozambique. Marine and Petroleum Geology 111, 179–201 (2020).
3. Deptuck, M.E., Steffens, G.S., Barton, M. & Pirmez, C. Architecture and evolution of upper fan channel-belts on the Niger Delta slope and in the Arabian Sea. Marine and Petroleum Geology 20, 649–676 (2003).
4. Posamentier, H.W. & Kolla, V. Seismic geomorphology and stratigraphy of depositional elements in deep-water settings. Journal of Sedimentary Research 73, 367–388 (2003).
5. Posamentier, H.W. Depositional elements associated with a basin floor channel levee system: case study from the Gulf of Mexico. Marine and Petroleum Geology 20, 677–690 (2003).
6. Mitchum Jr., R.M. Seismic stratigraphic expression of submarine fans. In: Berg, O.R., Woolverton, E.G. (Eds.), Seismic Stratigraphy II: an Integrated Approach to Hydrocarbon Exploration. In: AAPG Memoir 39. AAPG, Tulsa, pp.117–136 (1985).
7. Cant, D.J. Subsurface facies analysis. In, Walker, R.G. (Eds.), Facies models, Geoscience Canada Reprint Series 1, 297–310 (1984).
8. DSDP. Leg 25 Volcanogenic sediments and their relation to landmass volcanism and sea floor□ continent movements, Western Indian Ocean. Deep Sea Drilling Project Reports and Publications 25, 515–542, doi:10.2973/dsdp.proc.25.103.1974 (2007).
9. Velde, B., 1996. Compaction trends of clay-rich deep sea sediments. Marine Geology 133, 193–201.
10. Said, A., Moder, C., Clark, S. & Abdelmalak, M.M. Sedimentary budgets of the Tanzanian coastal basin and implications for uplift history of the East African rift system. Journal of African Earth Sciences 111, 288–295 (2015).
11. Sansom, P. A new stratigraphic model for Tanzania: Insights from deep water exploration. Presented at the Third EAGE Eastern Africa Petroleum Geoscience Forum, 7–9 November 2017, Maputo, Mozambique (2017).
12. Berggren, W.A. & Pearson, P.N. A revised (sub)tropical planktonic foraminiferal zonation of the Eocene and Oligocene. J. Foramin. Res. 35, 279–298 (2005).

13. Martini, E. Standard Tertiary and Quaternary calcareous nannoplankton zonation. Proc. 2nd Planktonic Conf. 2, 739–785, Rome, Italy (1971).
14. Wade, B.S., Pearson, P.N., Berggren, W.A. & Pälike, H. Review and revision of Cenozoic tropical planktonic foraminiferal biostratigraphy and calibration to the geomagnetic polarity and astronomical time scale. Earth-Science Reviews 104, 111–142 (2011).
